# Comparison of Different Cermet Coatings Sprayed on Magnesium Alloy by HVOF

**DOI:** 10.3390/ma14071594

**Published:** 2021-03-24

**Authors:** Ewa Jonda, Leszek Łatka, Wojciech Pakieła

**Affiliations:** 1 Department of Engineering Materials and Biomaterials, Silesian University of Technology, Konarskiego St. 18a, 44-100 Gliwice, Poland; wojciech.pakiela@polsl.pl; 2 Faculty of Mechanical Engineering, Wroclaw University of Science and Technology, Łukasiewicza St. 5, 50-371 Wroclaw, Poland; leszek.latka@pwr.edu.pl

**Keywords:** HVOF spraying, magnesium AZ31 alloy, microstructure, hardness, instrumented indentation, fracture toughness, wear resistance

## Abstract

In the present study, two different cermet coatings, WC–CrC–Ni and Cr_3_C_2_–NiCr, manufactured by the high-velocity oxy-fuel (HVOF) method were studied. They are labeled as follows: WC–CrC–Ni coating—WC and Cr_3_C_2_–NiCr coating—CrC. These coatings were deposited onto a magnesium alloy (AZ31) substrate. The goal of the study was to compare these two types of cermet coating, which were investigated in terms of microstructure features and selected mechanical properties, such as hardness, instrumented indentation, fracture toughness, and wear resistance. The results reveal that the WC content influenced the hardness and Young’s modulus. The most noticeable effect of WC addition was observed for the wear resistance. WC coatings had a wear intensity value that was almost two times lower, equal to 6.5·10^−6^ mm^3^/N·m, whereas for CrC ones it was equal to 12.6·10^−6^ mm^3^/N·m. On the other hand, the WC coating exhibited a lower value of fracture toughness.

## 1. Introduction

The development of modern technology forces a permanent search for structural solutions tending to the improvement of a product’s efficiency and quality, i.e., to the minimization of dimensions, an increase in reliability, and the maintenance of dimensional stability during its exploitation. Low density and relatively high strength represent material selection criteria for specific applications in industry. The materials that meet the abovementioned requirements include alloys of magnesium, titanium, and aluminum. The magnesium alloys, in addition to the combination of low density (1.7 g/cm^3^) [1] and high dimensional density, are also characterized by a good damping capacity, low casting shrinkage, good castability, and the possibility to apply them to the manufacturing of machinery and equipment that operate in temperatures reaching 300 °C. The application of magnesium alloys is not restricted to the automotive industry only, but also to the manufacturing of airplanes, computers, helicopters, home appliances, and office equipment as well as the chemical industry, aeronautics, radio engineering, and the power industry. A disadvantage of these materials is their low resistance to abrasive wear and corrosion. Alternative solutions to this issue include the application of surface engineering technologies to improve the applicative properties of the materials discussed herein [2,3,4,5].

The prospective solutions aimed at increasing the usable properties of magnesium alloys and, in consequence, the improvement of their applicative attractiveness include the methods of thermal spraying of coatings. The benefits resulting from the application of coatings include, without limitation, the possibility of regeneration and restoring the usable properties of machines and equipment that operate under conditions of abrasive, erosive, and corrosive wear as well as the combination of the beneficial properties of the core with resistance to wear, hardness, and heat. Depending on the source of heat used to melt the coating material, the following types of spraying can be differentiated: wire flame and powder flame spraying, crucible spraying, arc spraying, plasma spraying, and supersonic spraying [6,7,8]. The high-velocity oxygen fuel (HVOF) spraying method is one of the most commonly used methods. The high velocity of the powder particles and their moderate temperature cause them to “nest” on the surface of the sprayed substrate surface with immense adhesive force, enabling in effect the production of a very dense coating. The commercial powders for thermal spraying of hard metal coatings that are available on the market are based on hard materials (WC and Cr_3_C_2_). The most important binding metals are cobalt (Co) and nickel (Ni), which are frequently combined with chromium (Cr). Thus, in the case of an alloy binding agent, chromium is divided between the hard phase and the binder phase [9,10,11,12]. At present, the literature contains only a few papers related to the deposition of hard coatings on magnesium alloy substrates [12,13,14].

In this manuscript, the results of preliminary research are presented. Currently, there are several methods for producing coatings with the use of thermal spraying with the HVOF method with the use of commercial powders available on the market, including on structural alloyed and unalloyed quality steels for thermal improvement, stainless steels, and nickel alloys, while the use of light construction materials as the substrate in the form of magnesium alloys with low resistance to tribological factors has not been thoroughly researched and discussed to date.

## 2. Materials and Methods

### 2.1. Coating Deposition

**Figure 1:**
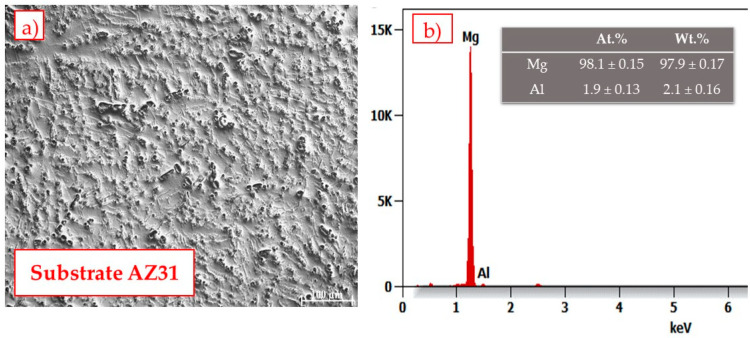
Cross-section image of the substrate (**a**) and the EDS analysis of the substrate (**b**).

Investigations were carried out on AZ31 magnesium alloy substrate samples. The diameter of the substrate was 100 mm and the thickness was 5 mm. The chemical composition of the alloy is shown in Figure 1. The particle size of the commercially available powders Cr_3_C_2_ 25% wt. NiCr (Amperit 588.059) and WC–CrC–Ni (Amperit 543.074), supplied by Höganäs, was in the range of 30 + 5 μm and 45 + 15 μm, respectively.

The chemical composition of the feedstock powders that were used in the coating manufacturing process is given in Table 1 [15].

**Figure 2:**
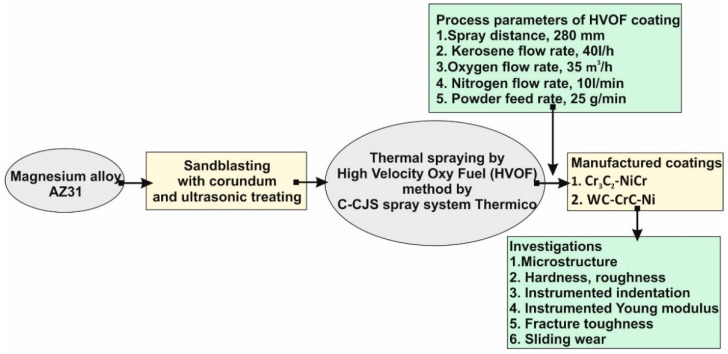
Schematic diagram of the manufacturing process and analysis method for WC and CrC coatings.

Figure 2 shows the scheme of the manufacturing process and investigation plan for the deposited WC–CrC–Ni (WC) and Cr_3_C_2_–NiCr (CrC) coatings. The surface of the magnesium alloy substrate was pre-processed by ultrasonic sandblasting with corundum. The corundum grit was F40, according to the FEPA (Federation of European Producers of Abrasives) standard. The C-CJS spray system Thermico (CERTECH Company, Wilamowice, Poland) was used to manufacture coatings. Kerosene and oxygen were used as the fuel media, whereas nitrogen was used as the carrier gas. During spraying, the maximum temperature of the flame was about 3250 K. The maximum temperature on the substrate’s surface was about 600 K. Some additional details can be found in [16].

### 2.2. Coating Characterization

#### 2.2.1. The Microstructure

The thermally sprayed coatings were analyzed using a scanning electron microscope (SEM, Supra 35, Zeiss, Oberkochen, Germany) with secondary electron and backscattered detectors. The observations were carried out on the coatings’ surfaces and their cross sections. The chemical composition was analyzed by energy dispersive X-ray spectroscopy (EDS).

#### 2.2.2. Surface Topography, Roughness, and Porosity

The surface roughness of sprayed coatings was measured by a stylus profilometer (MarSurf PS 10, Mahr, Germany), according to the ISO 4288 standard, with Gaussian filters according to the ISO 16610-21 standard [17]. Ten measurements on each sample were carried out. Afterwards, the average values, as well as standard deviations, were calculated for the R_a_ and R_z_ linear roughness parameters. Measurements of surface topography were carried out under ambient conditions and with the use of a commercial scanning probe. Coatings’ cross sections were observed by a Keyence VHX6000 (Keyence International, Mechelen, Belgium) microscope. Based on these images, carried out at 500× magnification, the porosity of sprayed coatings was estimated according to the ASTM E2109-01 standard [18]. Image J open-source software was used to calculate the porosity. Details of the porosity determination are given in [19].

#### 2.2.3. Mechanical Properties

The hardness of the coatings was tested on the ground and polished section by the Vickers hardness test method (HV0.5). The tests were performed along lines perpendicular to the specimen’s surfaces, along the run face axis. Hardness by instrumented indentation tests (HITs) were carried out on an NHT^3^ nanoindenter (Anton Paar, Graz, Austria) equipped with a Berkovich indenter and were performed at room temperature on the cross sections in accordance with the ISO 14577-4:2016 standard [20]. With this method, the instrumented Young’s modulus of the sprayed coatings was determined based on Oliver and Pharr’s methodology [21]. The details of the measurement procedure can be found in [22]. For hardness measurements, the value of the maximum load was equal to 500 mN, whereas for the elastic modulus (EIT) the range of maximum load was from 50 mN to 500 mN. In both cases, the dwell time was equal to 15 s. The fracture toughness of HVOF-sprayed coatings on magnesium alloy substrates was determined by a Vickers indentation test and cracks that occurred were measured according to the Palmqvist observation [23]. We next identified cracks according to the classification proposed and detailed by Chicot et al. [24]. There are two types of cracks, namely: (i) radial median ones; and (ii) Palmqvist ones. More information about these types can be found in [25]. In order to select the proper model for the indentation fracture toughness (IFT) determination, the ratio between total crack length (c) and half of the imprint diagonal (a) should be determined. When the c/a ratio is below 2.5 [26], the Palmqvist type of crack has formed. According to Chicot et al. [24] the formula for fracture toughness (*K_C_*) determination is as follows:

(1) $$K_{C\left(P\right)}=0.0089\cdot {\left(\frac{E}{H}\right)}^{2/5}\cdot \frac{P}{a\cdot l^{1/2}}$$

where:

*E*—Young modulus, MPa (obtained in the instrumented indentation test);

*H*—Vickers hardness, MPa;

*P*—maximum load in the indentation fracture toughness, N;

*a*—half of the imprint diagonal, m; and

*l*—average crack length, m.

The indentation toughness (IFT) was estimated with the Vickers indenter under the maximum load equal to 9.81 N (1 kG). Ten imprints in random locations on the coating cross sections were indented. In the literature, more than 30 different models of the IFT can be found [27]. For comparison with the results of the IFT values, some additional models were used in the current study: Niihara, Morena, Hasselman (NMH) [28] and Shetty, Wright, Mincer, and Clauer (SWMC) [29].

#### 2.2.4. The Wear Resistance

The wear resistance tests were carried out using the “pin-on-disc” method in accordance with the ASTM G99 [30] standard with linear mode tribometer version 6.1.19 (Anton Paar, Peseux, Switzerland). The test parameters are given in Table 2. To determine the mechanism of the wear, the topography was analyzed using an SEM and the wear rate (*K_V_*) was calculated from the wear formula of Lancaster [31]:

(2) $$K_{V}=\frac{V_{wear}}{F_{N}\cdot S}$$

where:

*V_wear_*—volume lost, mm^3^;

*F_N_*—normal load, N; and

*S*—sliding distance, m;

**Table 2:** The wear test parameters of the “pin-on-disc” method [30].

Linear Speed, cm/s	Temperature, °C	Test Frequency, Hz	Normal Load, N	Distance, m	Counter Body Al_2_O_3_, mm
5	20	2.65	10	50	6

## 3. Results and Discussion

### 3.1. The Microstructure

**Figure 3:**
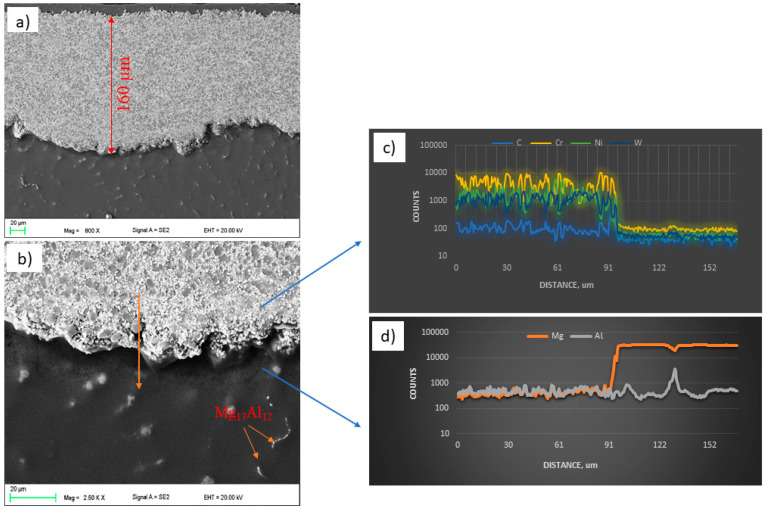
Structure (**a**,**b**) and results of the chemical analysis along the line from Figure b (**c**,**d**) of the WC coating.

**Figure 4:**
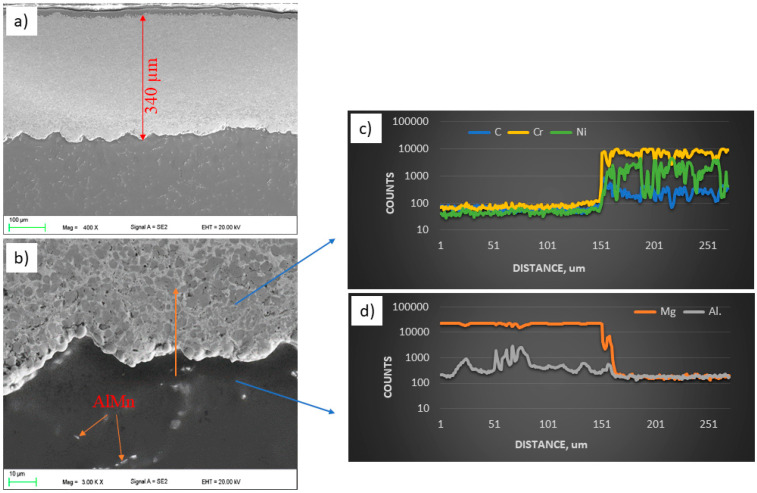
Structure (**a**,**b**) and results of the chemical analysis along the line from Figure b (**c**,**d**) of the CrC coating.

The observations of the WC and CrC microstructures of the coatings on the magnesium alloy substrate AZ31 using a scanning electron microscope were carried out in the BSD (backscatter electron detector) and SE (secondary electron) modes at an EHT (electron high tension) voltage from 5 to 20 kV. The EDS X-ray microanalysis revealed the presence of Mn (AlMn) and Al (Mg_17_Al_12_) phases in the substrate material in the entire volume. The microscopic observations did not reveal the surface treatment’s influence on the size and distribution of the intermetallic phases or changes in the AZ31 substrate’s structure (Figure 3b and Figure 4b).

The microstructures shown in Figure 3a and Figure 4a indicate uniform, compact, and layered coatings [32,33]. The average thickness of the coatings obtained as a result of HVOF spraying was about 162 ± 18 µm for WC and 344 ± 12 µm for CrC. The thickness of the WC coating was reduced when compared with CrC. Despite the fact that the spraying process was the same, the obtained thickness of the coatings was different. This may be because the tungsten carbide powder is heavier (2.5–3.5 g/cm^3^) than the Cr_3_C_2_–NiCr powder (2.3–3.0 g/cm^3^) according to the standard ASTM B212 [15]. Nevertheless, the lower deposition efficiency for the WC coating is related to the process parameters, which should be adjusted to the specific powder. We did not observe any discontinuity in the structure of the WC coating. In the case of the CrC coating, the pore size measured along the longest diagonal did not exceed 2 µm. The calculated porosity value for both samples was typical for HVOF coatings. For WC, it was equal to 3.7 ± 0.7 vol. % and for CrC it was equal to 2.4 ± 0.5 vol. %. The higher porosity of the WC coating may be due to the fact that its tungsten carbide (WC) powder has a high melting point (about 3143 K). Another factor that influenced the porosity value (which was higher for the WC sample than for the CrC one) is particle size. Similar values have been published by [34,35]. The occurrence of cracks and voids could result in a reduction in the durability of the coating as well as crack propagation and ultimately delamination. Moreover, the discontinuities in the structure reduce the resistance to hardness, erosion, and corrosion [36]. The linear chemical analysis of the coatings (Figure 3c and Figure 4c) and substrate (Figure 3d and Figure 4d), respectively, confirmed that there was no diffusion process between the coating and the substrate in both cases.

**Figure 5:**
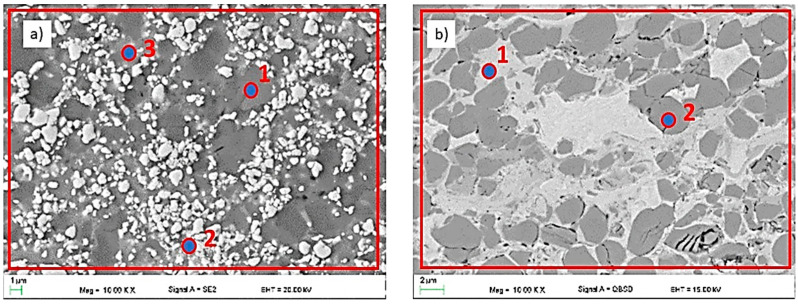
The microstructure of the (**a**) WC and (**b**) CrC coatings.

SEM micrographs and point element analysis as well as the area distribution showed spherical unmelted and semi-melted carbide particles (dark grey (Cr_3_C_2_) and white (WC) areas in Figure 5) in a metallic nickel matrix. A similar phenomenon was observed by Dent et al. [37].

An analogous phenomenon was observed for the CrC layer. Here, one can observe spherical, dark grey chromium carbides in the matrix of the bright nickel and chromium region. Black areas of irregular shape constitute porosity. The results of the chemical composition measurement are presented in Table 3 and Table 4. Both carbides were evenly distributed in the corresponding metal matrix. Evenly distributed structural elements, including carbides, significantly affect the strength of the coating. Sidhu et al. [34] came to similar conclusions. They combined the increase in the hardness of the obtained coatings with a high content of well-dispersed carbides in the matrix.

### 3.2. The Topography and Roughness of Coatings

**Figure 6:**
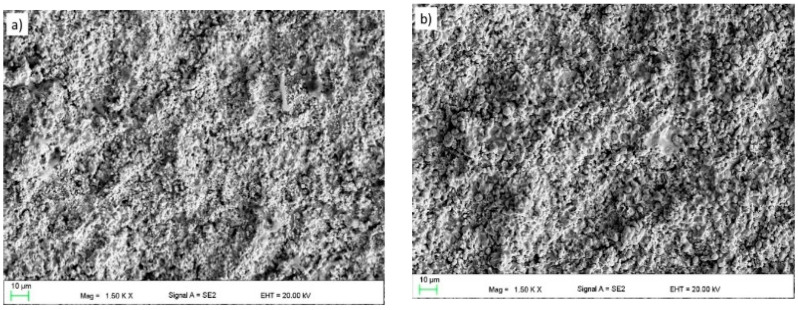
The topography of the thermally sprayed coating surface: (**a**) WC and (**b**) CrC (SEM).

The average surface roughness of the coatings in the as-sprayed condition is given in Table 5. The results are expressed as the average values from five measurements. The R_a_ (the arithmetic mean of ordinates of the roughness profile) and R_z_ (the maximum height of the roughness profile) roughness parameters were determined. It can be seen that all surface topography parameters of the WC coating are greater when comparing them to those of the CrC coating. We observed an increase of the roughness of about 33% between the two coatings. The higher surface roughness of the WC sample is connected to the bigger particles of the feedstock powder than for the CrC one. They are in the range of results reported in other papers for coatings with a similar chemical composition [38]. Figure 6a,b shows a topography of the manufactured coatings. In both cases, irregularly shaped grains of WC or Cr_3_C_2_ can be observed. This may be connected to the phenomenon that, in the flame, only the nickel matrix was melted, whereas the relatively big and hard grains were not dissolved [39].

### 3.3. The Hardness, Instrumented Indentation, and Fracture Toughness of the Coatings

**Figure 7:**
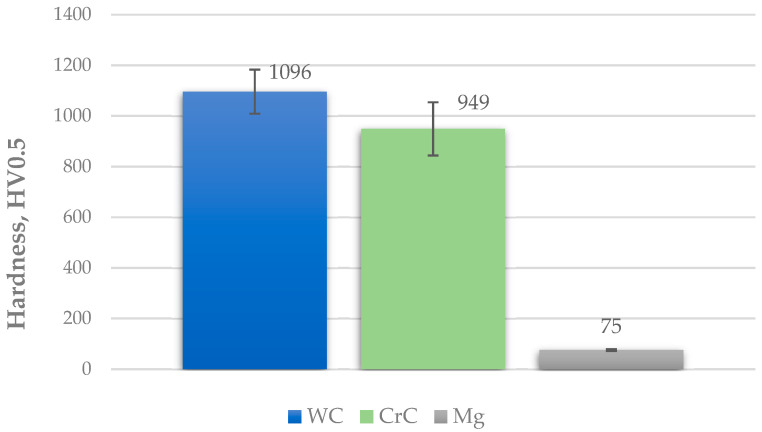
Comparison of average hardness values of the coated samples and substrate.

The hardness of the sprayed coatings increased significantly in comparison with the substrate. The average value of the WC coating is about 150 HV 0.5 higher than that of the CrC one. The average hardness of AZ31 alloy is 75 ± 4 HV 0.5 (Figure 7). It was reported in [40] that the hardness of the deposited CrC coating was the lower one and the main reason for the finding of hardness differences is the difference in their chemical composition. Additionally, [41] in their work indicate that coatings obtained by the HVOF method show higher hardness values comparatively with the substrate (about 74 ± 3 HV) [42]. The hardness of HVOF coatings increased due to the presence of Cr and W carbide phases in the structure. Similar values can be found in the literature [43].

The hardness instrumented indentation (HIT) tests were carried out in order to determine the microhardness and elastic modulus (EIT). An average of 10 measurements were taken. In the case of the WC coating, the estimated value of microhardness was higher and may be result of indentation in an area with more hard phases, while lower microhardness values will be found in areas with more binder phases or defects. Similar values can be observed in [44]. The obtained values of the hardness instrumented indentation and instrumented elastic modulus for the WC and CrC coatings are given in Table 6. When compared with other results in the literature, the obtained values of EIT are similar [45,46]. Slight differences could result in a different structure (mainly the porosity level) and different details of the phase composition, which are derivatives of the process parameters.

The obtained results confirm that the harder coating (WC) exhibited lower values of fracture toughness than the CrC coating. The average values as well as standard deviations of the fracture toughness of the manufactured composite coatings obtained from different models are shown in Table 7. As reported by [47], the CrC coating demonstrated a similar fracture toughness (4 ± 1 MPa m^1/2^). Similar results are also presented in [48].

### 3.4. Wear Resistance

Based on the sliding wear investigations, the manufactured coatings have good wear resistance. The values of wear rate were equal to 6.5 ± 1.4·10^−6^ mm^3^/(N·m) for the WC coating and 12.6 ± 3·10^−6^ mm^3^/(N·m) for the CrC coating.

**Figure 8:**
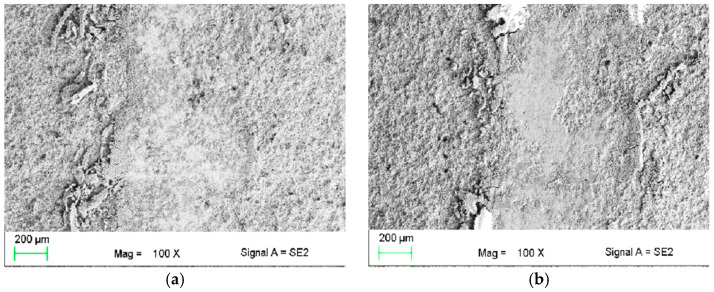
Wear tracks of the (**a**) WC and (**b**) CrC coatings, SEM.

The typical wear trace is given in Figure 8a,b. It indicates that the wear rate of the WC coating is lower than that of the CrC coating by about 50%. W. Fang et al. [49] reported that WC coatings are very protective for the substrate and have excellent wear resistance.

**Figure 9:**
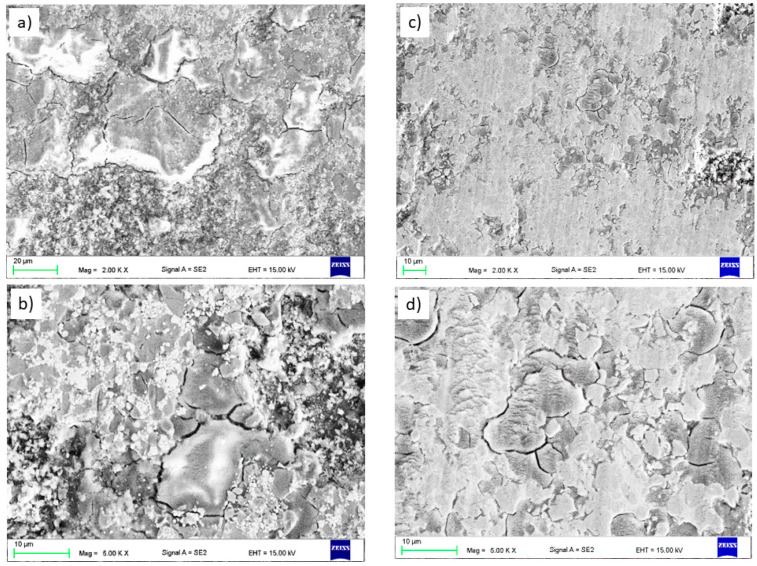
Details of wear tracks of manufactured coatings: (**a**,**b**) for the WC coating and (**c**,**d**) for the CrC coating, SEM.

**Figure 10:**
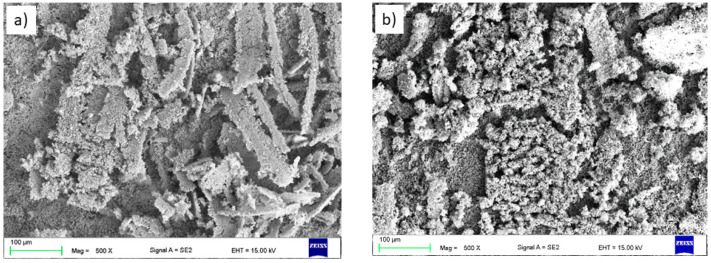
Wear debris observed at the edge of the wear track: (**a**) WC and (**b**) CrC coatings, SEM.

The abrasive wear resistance of thermally sprayed coatings depends on many factors, including microstructure, microhardness, and fracture toughness as well as cohesion in the coating [50]. A high abrasion resistance may depend on the coating having high hardness, which has also been reported in [51]. The wear resistance is also affected by the powder composition, so the WC coating displays better resistance. The mechanism that occurred is similar to the one observed and described by [52]. Additionally, [53] noticed that the thermally sprayed WC coating is a hard and wear-resistant material. The dominant mechanism of wear is a classic adhesive one. The increasing temperature during tests can cause oxidation wear to occur (bright areas in Figure 9). Much more oxidized areas were observed in the WC coating (Figure 9a,b). Analysis of the wear track on the WC coating revealed numerous cracks and evidence of the occurrence of fatigue and decohesion wear. In craters were observed small particles of wear debris. The occurrence of fatigue wear was also revealed during the observation of the wear debris (large flakes) (Figure 10a). In the wear track on the CrC coating, significantly fewer craters and signs of fatigue wear were observed. We also observed traces of low-cycle fatigue and cracks between individual hard Cr_3_C_2_ grains. The abrasion surface was much smoother than the that of the WC coating (Figure 9c). However, numerous places with plastic deformation of the surface were observed (Figure 9d), which is consistent with the values generally reported in the literature for such coatings tested under similar conditions [54]. Additionally, we also observed small traces of abrasive grooving in the matrix material. The wear debris observed at the edges of the wear track was finer than that of the WC coating (Figure 10b).

**Figure 11:**
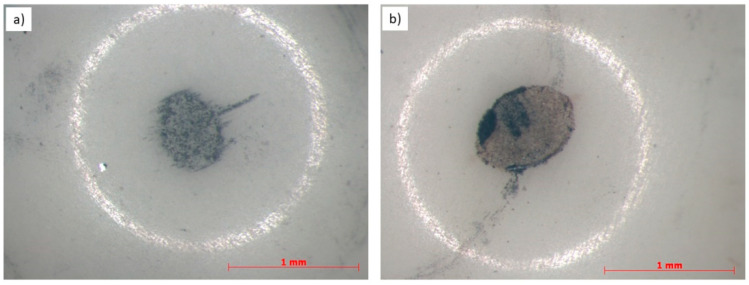
Comparison of Al_2_O_3_ counter-body wear traces after testing against the (**a**) WC and (**b**) CrC sprayed coatings.

In the case of the CrC coating, we observed some delamination and “wavy” regions, which are linked to the low-cycle fatigue. Probably, this is an effect of the microscale plastic deformation of the coating. This phenomenon was developed by Kato and Adachi [55]. For the WC coating, there were no low-cycle fatigue areas, but a crack net was clearly observed. This is connected to the higher hardness of the WC material than the CrC material and, consequently, there were higher stresses between the two abrasive bodies, which finally caused many cracks. It is a brittle cracking mode, as observed in [56]. The surface of the Al_2_O_3_ counter body after the wear resistance tests was quite clean and relatively smooth [57]; however, we also observed some shallow groves as an effect of the abrasive wear mode. There were interactions between the alumina, hard particles, and sometimes third-body oxidized debris particles (Figure 11a,b). A similar mechanism was observed in [56]. Additionally, the friction coefficient values were equal to 0.29 ± 0.02 and 0.65 ± 0.04 for the WC and CrC coatings, respectively. The obtained results are quite close to the values reported in the literature [58,59].

## 4. Conclusions

Based on the tests carried out on the WC and CrC coatings deposited on an AZ31 magnesium alloy substrate with HVOF spraying, the following conclusions were drawn:

1. The microstructures indicate uniform, compact, and layered coatings. The coating does not disclose cracks or voids. Despite the fact that the thermal spraying process of both coatings was performed with the same parameters, the coating thickness of the CrC is much greater than the coating thickness of the WC. This is the effect of the higher deposition efficiency and the lower powder density. The increased porosity of the WC coating is due to the higher melting point of the WC powder.
2. The increased roughness of the WC coating (Ra = 3.8 ± 0.3 µm) may be related to the poorer wettability of the substrate material AZ31 compared with the same relationship for the CrC coating (2.9 ± 0.2 µm).
3. The hardness of the sprayed coatings was equal to 1096 ± 87 and 949 ± 105 HV 0.5 for the WC and CrC coatings, respectively. In the case of hardness instrumented indentation (HIT), the estimated microhardness value of the WC coating was higher than that of the CrC coating, which may be due to an indentation in the area with hard phases, and the lower hardness of the coating may result from measurements taken in an area with more binder phases or defects.
4. The most noticeable effect of WC addition was observed for wear resistance. The WC coating showed a wear intensity value equal to 6.5·10^−6^ mm^3^/N·m, whereas for the CrC coating this value was equal to 12.6·10^−6^ mm^3^/N·m. As a result, it can be concluded that the WC coating has better wear resistance compared with the CrC coating. The better wear resistance of the WC coating results from the lower value of the friction coefficient (0.29 vs. 0.65) as well as the higher hardness (1096 HV0.5 vs. 949 HV0.5). On the other hand, the WC coating exhibited a slightly lower value of fracture toughness.

## Figures and Tables

**Table 1:** The chemical composition of the WC–CrC–Ni (WC) and and Cr_3_C_2_–NiCr (CrC) cermet powders [15].

Sample Code	Element, wt.%					
	Cr	C	Fe	Ni	O_2_	W
CrC	balance	9–11	max. 0.5	18–22	max. 0.6	–
WC	34–39	7.5–8.5	max. 0.3	14.5–17.5	max. 0.2	balance

**Table 3:** Results of the chemical composition analysis of the WC coating.

	Point 1 from Figure 5a		Point 2 from Figure 5a		Point 3 from Figure 5a		4—Area from Figure 5a	
	At %	Wt %	At %	Wt %	At %	Wt %	At %	Wt %
Cr	71 ± 0.1	51 ± 0.2	04 ± 0.2	11 ± 0.1	32 ± 0.6	43 ± 0.7	33 ± 0.2	32 ± 0.3
W	14 ± 0.4	02 ± 0.1	94 ± 0.1	70 ± 0.1	29 ± 0.4	11 ± 0.1	42 ± 0.4	11 ± 0.7
Ni	–	–	–	–	38 ± 0.1	45 ± 0.2	14 ± 0.1	12 ± 0.1
C	14 ± 0.1	45 ± 0.1	01 ± 0.7	18 ± 0.8	–	–	10 ± 0.4	43 ± 0.9

**Table 4:** Results of the chemical composition analysis of the CrC coating.

	Point 1 from Figure 5b		Point 2 from Figure 5b		3—Area from Figure 5b	
	At %	Wt %	At %	Wt %	At %	Wt %
Cr	34 ± 4.3	37 ± 2.1	84 ± 5.8	56 ± 1.2	59 ± 2.2	42 ± 0.6
Ni	65 ± 2.7	62 ± 3.9	–	–	28 ± 4.1	17 ± 0.9
C	–	–	15 ± 2.2	43 ± 0.8	12 ± 0.7	39 ± 3.5

**Table 5:** The average surface roughness of the coatings in the as-sprayed condition.

Sample Code	Roughness Parameter	
	R_a_, µm	R_z_, µm
WC	3.8 ± 0.3	22.1 ± 1.3
CrC	2.9 ± 0.2	13.3 ± 0.9

**Table 6:** The mechanical properties of the coatings in the as-sprayed condition.

Sample Code	HIT, GPa	EIT, GPa
WC	11.8 ± 1.7	314
CrC	8.7 ± 0.5	224

**Table 7:** Indentation fracture toughness of composite coatings sprayed by the high-velocity oxygen fuel (HVOF) method.

Sample Code	Indentation Fracture Toughness, MPa·m^1/2^		
	Palmqvist	NMH	SWMC
WC	3.8 ± 0.5	3.6 ± 0.6	3.6 ± 0.6
CrC	4.5 ± 0.8	4.4 ± 0.7	4.6 ± 0.7

## Data Availability

The data presented in this study are available on request from the corresponding author. The data presented in this manuscript will be available in the scientific publication repository of the Silesian University of Technology.

## References

[1] Kulekci M.K. (2008). Magnesium and its alloys applications in automotive industry. Int. J. Adv. Manuf. Technol..

[2] Tański T. (2012). Shaping the structure and surface of Mg-Al-Zn alloys. Sci. Int. J. World Acad. Mater. Manuf. Eng..

[3] Dziadoń A. (2012). Magnesium and Its Alloys. Monographs.

[4] Fouad Y., El Batanouny M. (2011). Effect of surface treatment on wear behavior of magnesium alloy AZ31. Alex. Eng. J..

[5] Lin B., Zhang B., Ma M., Zhang K., Li Y., Chen Z., Liu Y. (2020). Optimization of Plasma Spraying for VW75 Rare Earth Magnesium Alloy Based on Orthogonal Experiments and Research on Its Performances. Coatings.

[6] Tejero-Martin D., Rezvani Rad M., McDonald A., Hussain T. (2019). Beyond traditional coatings: A review on thermal-sprayed functional and smart coatings. J. Spray Technol..

[7] La Barbera-Sosa J.G., Santana Y.Y., Caro J., Chicot D., Lesage J., Staia M.H., Puchi-Cabrera E.S. (2014). Mechanical properties of WC coatings evaluated using instrumented indentation technique. Surf. Eng..

[8] Janka L. (2018). Thermally Sprayed Cr_3_C_2_-NiCr Coatings-Improving the Abrasion Resistance. Ph.D. Thesis.

[9] Zheng C., Liu Y., Qin J., Chen C., Ji R. (2017). Wear behavior of HVOF sprayed WC coating under water-in-oil fracturing fluid condition. Tribol. Int..

[10] Picas J.A., Punset M., Rupérez E., Menargues S., Baile M.T. (2019). Corrosion mechanism of HVOF thermal sprayed WC-Co-Cr coatings in acidic chloride media. Surf. Coat. Technol..

[11] Zdravecka E., Suchanek J., Tkacova J., Trpcevska J., Brinkienė K. (2010). Investigation of wear resistance of high velocity oxy-fuel sprayed WC-Co and Cr_3_C_2-_NiCr coatings. Mechanics.

[12] Parco M., Zhao L., Zwick J., Bobzin K., Lugscheider E. (2006). Investigation of HVOF spraying on magnesium alloys. Surf. Coat. Technol..

[13] Pokhmurska H., Wielage B., Lampke T., Grund T., Student M., Chervinska N. (2008). Post-treatment of thermal spray coatings on magnesium. Surf. Coat. Technol..

[14] Jonda E., Łatka L., Pakieła W. (2020). Microstructure and selected properties of Cr_3_C_2_-NiCr coatings obtained by HVOF on magnesium alloy substrates. Materials.

[15] Höganäs.com Powder Technologies. Surface Coating. https://www.hoganas.com/globalassets/download-media/stc/pd-4057.pdf.

[16] Jonda E., Łatka L., Więcław G. (2020). Preliminary Studies on HVOF Sprayed Coatings on Magnesium Alloys. Mater. Process..

[17] ISO16610-21: 2011 Geometrical Product Specification (GPS)–Filtration–Part 21: Linear Profile Filteres: Gaussian Filteres. https://www.iso.org/standard/50176.html.

[18] ASTM E2109-01 (2014). Standard Test Methods for Determining Area Percentage Porosity in Thermal Sprayed Coatings.

[19] Michalak M., Łatka L., Szymczyk P., Sokołowski P. (2017). Computational image analysis of Suspension Plasma Sprayed YSZ coatings. Proceedings of the ITM Web of Conferences, II International Conference of Computational Methods in Engineering Science (CMES’17).

[20] ISO 14577-4 (2016). Metallic Materials–Instrumented Indentation Test for Hardness and Materials Parameters–Part 4: Test Method for Metallic and Non-Metallic Coatings.

[21] Oliver W.C., Pharr G.M. (1992). An improved technique for determining hardness and elastic modulus using load and displacement sensing indentation experiments. J. Mater. Res..

[22] Łatka L., Chicot D., Cattini A., Pawłowski L., Ambroziak A. (2013). Modeling of elastic modulus and hardness determination by indentation of porous yttria stabilized zirconia coatings. Surf. Coat. Technol..

[23] Palmqvist S. (1962). Occurrence of crack formation during Vickers indentation as a measure of the toughness of hard materials. Arch. Eisenhuettenwes.

[24] Chicot D., Duarte G., Tricoteaux A., Jorgowski B., Leriche A., Lasage J. (2009). Vickers indentation fracture (VIF) modeling to analyze multi-cracking toughness of titania, alumina and zirconia plasma sprayed coatings. Mater. Sci. Eng. A.

[25] Lube T. (2001). Indentation crack profiles in silicon nitride. J. Eur. Ceram. Soc..

[26] Roy M., Pauschitz A., Bernardi J., Koch T., Franek F. (2006). Microstructure and mechanical properties of HVOF sprayed nanocrystalline Cr_3_C_2_-25(Ni20Cr) coating. J. Spray.

[27] Ponton C.B., Rawlings R.D. (1989). Vickers indentation fracture toughness test Part 1 Review of literature and formulation of standardised indentation toughness equations. Mater. Sci. Technol..

[28] Niihara K., Morena R., Hassleman D.P.H. (1982). Evaluation of K_IC_ of brittle solids by the indentation method with low crack to indent ratio. J. Mater. Sci. Lett..

[29] Shetty D.K., Wright I.G., Mincer P.N., Clauer A.H. (1985). Indentation fracture of WC-Co cermets. J. Mater. Sci..

[30] ASTM G99-17 (2005). Standard Test Method for Wear Testing with a Pin-on-Disk Apparatus.

[31] Lancaster J.K. (1967). The influence of substrate hardness on the formation and endurance of molybdenum disulphide films. Wear.

[32] Lima R.S., Karthikeyan J., Kay C.M., Lindemann J., Berndt C.C. (2002). Microstructural characteristics of cold-sprayed nanostructured WC. Co coatings. Thin Solid Film..

[33] Guilemany J.M., Fernández J., Delgado J., Benedetti A.V., Climent F. (2002). Effects of thickness coating on the electrochemical behavior of thermal spray Cr3C2-NiCr coatings. Surf. Coat. Technol..

[34] Sidhu H.S., Sidhu B.S., Prakash S. (2006). Mechanical and microstructural properties of HVOF sprayed WC-Co and Cr3C2-NiCr coatings on the boiler tube steels using LPG as the fuel gas. J. Mater. Process. Technol..

[35] Zhan S.-H., Cho T.-Y., Yoon J.-H., Li M.-X., Shum P.W., Kwon S.-C. (2009). Investigation on microstructure, surface properties and anti-wear performance of HVOF sprayed WC-Cr-Ni coatings modified by laser heat treatment. Mater. Sci. Eng. B.

[36] Espalllargas N., Berget J., Guilemany J.M., Suegama A.V. (2008). Cr3C2-NiCr and WC-Ni thermal spray coatings as alternatives to hard chromium for erosion-corrosion resistance. Surf. Coat. Technol..

[37] Dent A.H., Horlock A.J., Mc Cartney D.G., Harris S.J. (2000). Microstructure formation in high velocity oxy–fuel thermally sprayed Ni-Cr-Mo-B alloys. Mater. Sci. Eng..

[38] Şerban V.A., Uţu I.D., Mărginean G. (2015). Substrate influence on the properties of thermally sprayed WC-CrC-Ni cermet coatings. J. Optoelectron. Adv. Mater..

[39] Ding X., Ke D., Yuan C., Ding Z., Cheng X. (2018). Microstructure and Cavitation Erosion Resistance of HVOF Deposited WC-Co Coatings with Different Sized WC. Coatings.

[40] Richert M.W. (2011). The wear resistance of thermal spray the tungsten and chromium carbides coatings. J. Achiev. Mater. Manuf. Eng..

[41] Murariu A.C., Pleşu N., Perianu I.A., Tară-Lungă-Mihali M. (2017). Investigations on corrosion behavior of WC-CrC-Ni coatings deposited by HVOF Thermal Spraying Process. Int. J. Electrochem. Sci..

[42] Serindag H.T., Kiral B.G. (2017). Friction Stir Welding of AZ31 Magnesium Alloys—A Numerical and Experimental Study. Lat. Am. J. Solids Struct..

[43] Buzdugan R.M., Murariu A.C., Perianu I.-A., Simon N. (2016). Abrasive wear resistance of HVOF thermal sprayed WC-CrC-Ni coatings. Weld. Mater. Test..

[44] Qiao L., Wu Y., Hong S., Long W., Cheng J. (2021). Wet abrasive wear behavior of WC-based cermet coatings prepared by HVOF spraying. Ceram. Int..

[45] Houdkova S., Blahova O., Zahalka F., Kasparova M. (2012). The Instrumented Indentation Study of HVOF-Sprayed Hardmetals Coatings. J. Spray Technol..

[46] Matikainena V., Peregrina S.R., Ojala N., Koivuluoto H., Schubert J., Houdková Š., Vuoristo P. (2019). Erosion wear performance of WC-10Co4Cr and Cr_3_C_2_-25NiCr coatings sprayed with high-velocity thermal spray processes. Surf. Coat. Technol..

[47] Amudha A., Nagaraja H.S., Shashikala H.D. (2021). Mechanical and wetting properties of 25%NiCr-75%Cr_2_C_3_ cermet coated on low carbon steel using HVOF thermal spray technique. Phys. B Condens. Mater..

[48] Yao H.-L., Yang C., Yi D.-L., Zhang M.-X., Wang H.-T., Chen Q.-Y., Bai X.-B., Ji G.-C. (2020). Microstructure and mechanical property of high velocity oxy-fuel sprayed WC-Cr_3_C_2_-Ni coatings. Surf. Coat. Technol..

[49] Fang W., Cho T.Y., Yoon J.H., Song K.O., Hur S.K., Youn S.J., Chun H.G. (2009). Processing optimization, surface properties and wear behavior optimization. Process. Technol..

[50] Xie M., Zhang S., Li M. (2013). Comparative investigation on HVOF sprayed Carbide-based coatings. Appl. Surf. Sci..

[51] Karaoglanli A.C., Oge M., Doleker K.M., Hotamis M. (2017). Comparison of tribological properties of HVOF sprayed coatings with different composition. Surf. Coat. Technol..

[52] Xu H.S., Dong Q. (2020). Conference Series: Friction and wear behavior of WC-Ni coatings HVOF sprayed on duplex stainless steel. Mater. Sci. Eng..

[53] Huang T.-S. (2011). Microstructure and Properties of Thermal Sprayed WC-CrC-Ni Coatings. China Steel Tech. Rep..

[54] Bolelli G., Berger L.-M., Börner T., Koivuluoto H., Matikainen V., Lusvarghi L., Lyphout C., Markocsan N., Nylén P., Sassatelli P. (2016). Sliding and abrasive wear bahavior of HVOF- and HVAF-sprayed Cr_3_C_2_-NiCr hardmetals coatings. Wear.

[55] Kato K., Adachi K. (2002). Wear of advanced ceramics. Wear.

[56] Bolelli G., Berger L.-M., Bonetti M., Lusvarghi L. (2014). Comparative study of the dry sliding wear behavior of HVOF-sprayed WC-(W,Cr)2C-Ni and WC-CoCr hardmetals coatings. Wear.

[57] Michalak M., Łatka L., Sokolowski P., Toma F.-L., Myalska H., Denoirjean A., Ageorges H. (2020). Microstructural, mechanical and tribological properties of finely grained Al_2_O_3_ coatings obtained by SPS and S-HVOF methods. Surf. Coat. Technol..

[58] Bhosal D.G., Prabhu T.R., Rathod W.S. (2020). Sliding and erosion wear behavior of thermal sprayed WC-Cr_3_C_2_-Ni coatings. Surf. Coat. Technol..

[59] Mohanty M., Smith R.W., De Bonte M., Celis J.P., Lugscheider E. (1996). Sliding wear behavior of thermally sprayed 75/25 Cr_3_C_2_/NiCr wear resistant coatings. Wear.

